# Vascular Calcification Mechanisms: Updates and Renewed Insight into Signaling Pathways Involved in High Phosphate-Mediated Vascular Smooth Muscle Cell Calcification

**DOI:** 10.3390/biomedicines9070804

**Published:** 2021-07-12

**Authors:** Nima Abbasian

**Affiliations:** School of Life and Medical Sciences, University of Hertfordshire, Hertfordshire AL10 9AB, UK; abbasian.n.174@gmail.com

**Keywords:** vascular calcification, phosphate, signaling, CKD, microRNAs, DNA damage, senescence, epigenetic

## Abstract

Vascular calcification (VC) is associated with aging, cardiovascular and renal diseases and results in poor morbidity and increased mortality. VC occurs in patients with chronic kidney disease (CKD), a condition that is associated with high serum phosphate (Pi) and severe cardiovascular consequences. High serum Pi level is related to some pathologies which affect the behaviour of vascular cells, including platelets, endothelial cells (ECs) and smooth muscle cells (SMCs), and plays a central role in promoting VC. VC is a complex, active and cell-mediated process involving the transdifferentiation of vascular SMCs to a bone-like phenotype, systemic inflammation, decreased anti-calcific events (loss of calcification inhibitors), loss in SMC lineage markers and enhanced pro-calcific microRNAs (miRs), an increased intracellular calcium level, apoptosis, aberrant DNA damage response (DDR) and senescence of vascular SMCs. This review gives a brief overview of the current knowledge of VC mechanisms with a particular focus on Pi-induced changes in the vascular wall important in promoting calcification. In addition to reviewing the main findings, this review also sheds light on directions for future research in this area and discusses emerging pathways such as Pi-regulated intracellular calcium signaling, epigenetics, oxidative DNA damage and senescence-mediated mechanisms that may play critical, yet to be explored, regulatory and druggable roles in limiting VC.

## 1. Introduction

**Figure 1:**
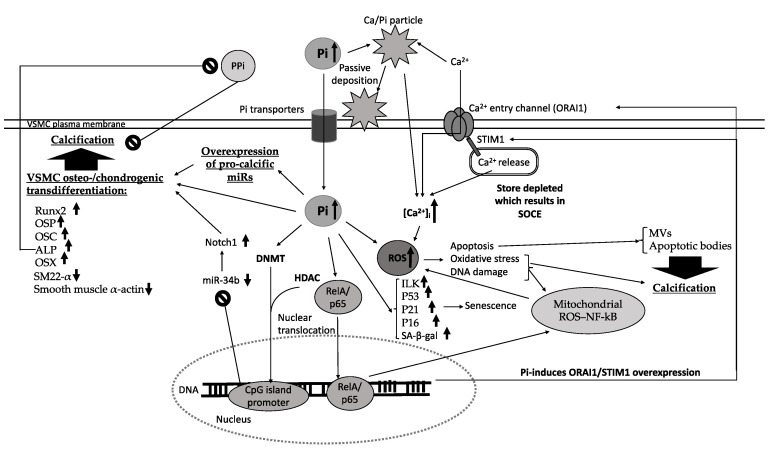
Schematic illustrating the major mechanisms involved in high Pi-induced vascular calcification (VC). VC is an active cell-mediated process whereby Vascular Smooth Muscle Cells (VSMCs) play a central role. In response to calcifying inducers, of note high serum phosphate (Pi), VSMCs undergo osteo-/chondrogenic transdifferentiation which renders contractile VSMCs to become a bone-resembling phenotype. As will be discussed in Section 2, Section 3, Section 4, Section 5, Section 6 and Section 7 of the review, transdifferentiated bone-like VSMCs actively promote VC which results in an increased risk of cardiovascular mortality. This process includes signaling pathways that induce loss of calcification inhibitors such as pyrophosphate (PPi) and overexpression of the osteogenic transcription factors including runt-related transcription factor 2 (Runx2), osteopontin (OSP), osteocalcin (OSC), alkaline phosphatase (ALP), and osterix (OSX). This process may also be partly mediated by some emerging novel signaling mechanisms, yet to be fully explored. Briefly, these include high Pi-mediated cellular senescence, oxidative DNA damage, an increase in intracellular calcium levels, altered pro-calcific microRNAs (miRs), and epigenetic factors. ROS: reactive oxygen species; MVs: matrix vesicles; STIM1: stromal interaction molecule 1; ORAI1: calcium release-activated calcium channel protein 1; SOCE: store operated calcium entry; ILK: integrin linked kinase; senescence-associated β-galactosidase; DNMT: DNA methyltransferases; HDAC: histone deacetylase; CpG: cytosine phosphate-guanine.

Vascular calcification (VC) is a feature characteristic of normal arterial aging [1] but is also associated with cardiovascular [2] and renal [3,4,5,6] diseases with poor prognosis and increased mortality. VC occurs in patients with chronic kidney disease (CKD), a condition that is associated with high serum phosphate (Pi) levels (known as hyperphosphatemia) and severe cardiovascular consequences [7]. Hyperphosphatemia occurs when there is an impairment in Pi homeostasis; strikingly, this happens in patients with CKD [7]. Furthermore, even though this remains to be fully addressed, other disorders may also result in an increase in Pi, such as hypoxia and ischemia [8]. This is also true in cancer patients with acute tumor lysis syndrome following chemotherapy [9], within the tumor microenvironment [10] and in the premature aging presented in klotho (-/-) mice [11]. The importance of Pi homeostasis in CKD and regulation of VC has been recognised for decades, but novel insights, which are relevant to Pi signaling regulation of VC, continue to emerge. Even though VC was originally thought to be a passive process of ectopic deposition of calcium/phosphate minerals within the vascular wall as a result of a net positive calcium and phosphate levels in the blood, it is now well-documented that VC is an active cell-mediated process involving transdifferentiation of smooth muscle cells (SMCs) into bone-like phenotypes [12]. It may occur at different sites within the cardiovascular system including arteries, valves, and the tunica intima and media [13,14,15,16]. In patients with renal disease, VC may occur in both the media and intima [6], albeit the most common type has been shown to be medial calcification [15]. Medial calcification occurs as a consequence of local inflammation and transdifferentiation of vascular SMC (VSMC) into an osteoblast-like phenotype which *per se* is a consequence of imbalanced serum calcium and phosphate metabolism [5,16,17]. In comparison, intimal calcification occurs secondary to atherosclerosis and is observed alongside inflammation and lipid/cholesterol deposition [18]. Once progressed to an advanced level, VC can promote poor clinical outcomes including aortic stiffening, aortic valve stenosis or occlusive lesions as seen alongside atherosclerotic plaques [4,14,18]. This review briefly discusses the mechanisms mediating VC and highlights the role that an excess Pi milieu plays in promoting VC (Figure 1). The current understanding of the mechanisms of Pi-mediated VC is reviewed; subsequently, what is known about the possible contribution of Pi-mediated epigenetic regulation of VC, Pi-dependant regulation of intracellular calcium signals, oxidative stress, cellular senescence and the aberrant DNA damage response in regulating VC and some of directions for future research in this area are discussed. The contribution of microRNAs (miRs) in mediating calcification of SMC in response to a high Pi milieu is also reviewed. Clarification of the mechanisms mediating VC may lead to the development of new therapeutic strategies to prevent, if not reverse, calcification in disease states such as CKD.

## 2. Mechanisms of VC

VSMCs, derived from mesenchymal stem cells (MSCs), can transdifferentiate into other cells of mesenchymal origins when under cellular stress, such as cells of the mesodermal lineage, including bone and cartilage cells (of note osteoblasts and chondrocytes) [19]. VC is characterized by the osteogenic transformation of VSMC [20]; this includes loss of smooth muscle cells’ lineage markers (e.g. SM22-α and smooth muscle α-actin) and the gaining of osteogenic markers, including: overexpression of transcription factor runt-related transcription factor 2 (Runx2), which is the master regulator of osteoblastic differentiation; and increased DNA-binding activity of the transcription factor core binding factor alpha1 (Cbfa1) and genes containing the Cbfa1 binding site including osteopontin (OSP), osteocalcin (OSC), and alkaline phosphatase (ALP) [20]. The inhibitory enzymatic activity of inorganic pyrophosphate (PPi), an important endogenous inhibitor of VC [21], is significantly abrogated by an increase in ALP activity [22]. The transdifferentiation of VSMC to bone-like phenotypes (i.e., osteo-/chondroblast-like cells) further becomes exacerbated with the induction of oxidative stress, detective DNA damage response (DDR), cellular senescence, apoptosis, the release of extracellular matrix vesicles (EVs) (particularly exosomes), pro-calcific microRNAs (miRs) and elastin degradation, which all result in the establishment of mineralisation nodules promoting calcification [23,24,25]. Even though there are an overwhelming number of studies on the association of risk factors such as hyperphosphatemia, hypercalcemia, oxidative stress, inflammation, and apoptosis in promoting VC, there is a lack of clarity on the precise contribution of some of the newest and novel regulatory pathways and molecules in promoting VC. These include elevated level of EVs, pro-calcific miRs (either free, protein-bound or within EVs), defective DDR, cell senescence, epigenetics, and the possible interplay between these factors (e.g., potential cross-talk between hyperphosphatemia, epigenetic factors, intracellular calcium levels, oxidative stress, DNA damage, defective DDR and cell senescence). To correctly fit these into the VC pathogenesis map, this requires further intensive investigations.

OSP is a phosphorylated glycoprotein and a key component in bone development. It has a conserved cell-binding sequence (Arg-Gly-Asp (RGD)) that binds calcium and is chemotactic and adhesive for rat VSMCs [26]. It is known that the expression level of OSP at both the RNA and protein level is controlled by the enzymatic activity of ALP, which results in the generation of free Pi in the extracellular milieu. This is a Pi signal that is necessary for the induction of OSP mRNA and protein levels [27]. Given the well-known inhibitory role that Pi plays in inhibiting the catalytic activity of phosphatases [28,29,30,31], it is possible, but to be precisely determined, that ALP and Pi may work in a co-regulatory on one another, meaning that the enzymatic activity of ALP enhances the release of Pi and freed Pi then inhibits the activity of ALP. Even though the OSP protein is strongly associates with calcification in vivo, a previous study showed that human VSMCs express little OSP when compared with rat VSMCs, indicating that there is a remarkable difference in the biology of human and rodent VSMCs [26]. Compared to monolayer cultures, it has been shown that human VSMCs spontaneously form multicellular nodules and deposit calcium crystals. It has also been shown that the cells in calcifying nodules expresses higher level of the SMC markers α-SM actin, SM22α, and calponin and increased levels of matrix Gla protein (MGP) mRNA alongside barely detectable OSP mRNA and protein levels, [32] indicating that OSP is not an essential factor for the initiation and development of calcification [32]. Another study examining the pattern of response to the calcifying milieu (via cells treated with sodium orthophosphate, β-glycerophosphate or β-glycerophosphate/sodium diphosphate) compared VSMCs with osteoblasts. This study found that VSMCs, in vitro, respond to calcification inducers by a remarkably lower order of magnitude in up-regulating mRNA expression of osteoblast-related genes (e.g. Runx-2, osterix (OSX), OSC, OSP) compared to bone-forming osteoblasts [12]. Treatment of bovine aortic smooth muscle cells (BASMC) with β-glycerophosphate for ten days resulted in an increase in expression and DNA-binding activity of the transcription factor Cbfa1 and genes containing the Cbfa1 binding site, including OSP, OSC, and ALP, as well as a decrease in SM22-α and smooth muscle α-actin [20]. Similarly, another study reported that treatment of bovine vascular smooth muscle cells (BVSMC) with β-glycerophosphate or uremic sera resulted in an increase in OSP expression and ALP activity in BVSMC [33]. Blocking sodium-dependant Pi co-transport or ALP activity partially inhibited uremic sera-induced OSP expression [33], indicating that even though mineral deposition in BVSMC induced by incubation with uremic sera is partially regulated by Pi level, it also depends on other factors such as imbalanced levels of fibroblast growth factor 23 (FGF-23), parathyroid hormone (PTH) and other serum pro-calcific biomarkers. However, it is of note that the inhibitor of the Pi transporter utilized in the earlier study [33], foscarnet (also known as PFA or Sodium phosphonoformate tribasic hexahydrate), a structural analogue of Pi in which one hydroxyl group is replaced with a carboxyl group, is a weak inhibitor for sodium-dependent Pi transporter in VSMCs [34]. Therefore, the earlier study could benefit from a more specific sodium-dependant Pi co-transporter which could potentially result in a greater level of inhibition of, or fully inhibit, the observed uremic sera-induced OSP expression [33] which needs to be reassessed in future research endeavours.

It has been shown that the incubation of human arterial SMCs (hAVSMCs) with uremic serum induced calcification, a process mediated by overexpression of bone morphogenetic protein (BMP-2) and Runx-2 in SMC [35]. This suggested a process whereby serum from disease settings is evidently primed to promote calcification even outside of the disease milieu. In an earlier study investigating the effect of uremic serum on bovine VSMCs, it was shown that uremic serum induces overexpression of Runx-2, OSC, and increased ALP activity in a cyclic AMP (cAMP)/protein kinase A (PKA), but not protein kinase C, dependant signaling pathway [36]. It has also been shown that uremic serum contains an increased BMP-2 concentration. Noggin, an inhibitor of BMP, decreased Runx-2 expression [36]. It remains to be fully clarified, however, what biomarker(s) in patients’ serum initiate calcification both in vitro and in vivo. Meanwhile, a net positive calcium and phosphate and deranged levels of phosphate-responsive hormones (i.e. phosphatonins) may partly explain how incubating hAVSMCs with uremic serum induced calcification in the earlier study [35] but identification of a full panel of serum biomarkers capable of inducing calcification remains an open research question.

## 3. Pi Regulation of VC

High serum Pi is frequently observed in patients with CKD [37]. Pi overload affects the behaviour of vascular cells, including endothelial cells (ECs) [28,29,38,39], SMCs [16,40,41,42] and platelets [43]. Excess Pi concentration has reportedly been shown to induce SMC osteo-/chondrogenic transdifferentiation [44,45,46]. In a previous study consisting of patients with coronary artery disease (CAD) with or without end-stage renal failure (ESRF), it has been found that within the lesions of coronary plaques in subgroups with ESRF there is the occurrence of more calcified plaques compared to controls (CAD without renal insufficiency), where fibroatheroma and atheroma found to be more frequent [47]. It has also been observed that in CAD patients with renal failure, the tunica media but not intima becomes thickened, and calcified plaques are mainly composed of hydroxyapatite and CaPi rather than calcium oxalate crystals [47]. In cultured ECs, a higher concentration of Pi acts intracellularly as an inhibitor of cellular phosphoprotein phosphatases (of note phosphotyrosine phosphatases [29] and at least one of the major cellular phosphoserine/threonine phosphatases; i.e. PP2A [28]) which results in enhanced phosphorylation signals and cell activation culminating in cytoskeletal changes [28,29] with an endpoint of pro-coagulant plasma microvesicles released from ECs both in vitro [29,39] and in vivo [48]. In human aortic valvular interstitial cells, inhibition of PP2A promotes osteogenic differentiation of the cells via extracellular signal-regulated kinase (ERK) and the p38 MAPK pathway [49]. In VSMCs, a Pi load has been reported to activate ERK and the mammalian target of rapamycin (mTOR), which were required for VC [41]. It remains to be determined if high extracellular Pi on VSMCs can result in an increase in intracellular Pi levels and initiate osteogenic differentiation of SMCs via inhibition of PP2A coupled ERK and the p38 MAPK signaling pathway.

Polyphosphate (polyP) is an inorganic polymer of linear-linked orthophosphate residues. Previous studies have pointed out the important role of extracellular polyP in the prevention of VC both by hydrolysing to PPi and also by acting directly as an inhibitor of calcium phosphate deposition (CPD) [21,50]. Given the emerging role that Pi may play in regulating polyP content in mammalian cells [43], it now merits further investigation whether Pi-mediated accumulation in intracellular polyP and conversely depletion of polyP in the extracellular milieu could contribute to the signaling map of Pi-mediated VC.

## 4. Pi-Mediated Signaling Pathways

### 4.1. Oxidative Stress

Oxidative stress occurs when the production of reactive oxygen species (ROS), by-products of aerobic metabolism, exceeds antioxidant defences. VC is an active cell-mediated process that occurs in response to oxidative stress, inflammatory cytokines, extracellular matrix deposition and increased calcium and phosphate levels [16,51,52]. Oxidative stress has been reported to be an important mediator of VSMCs’ osteochondrogenic transdifferentiation and is closely associated with the development of VC in vitro and in vivo in patients with CKD [45,51,53,54]. Previous studies have shown that high concentrations of Pi can induce oxidative stress and VSMC calcification [55]. This is a Pi effect which could be inhibited by activation of the KEAP1 (Kelch-like ECH-associated protein 1)/NF-E2-related factor 2 (NRF2)/P62 antioxidative pathway [55]. Nuclear factor-κB (NF-κB), expressed in nearly all mammalian cells, is an inducible transcription factor which is involved in cell adhesion, proliferation, differentiation, autophagy, and protection against apoptosis and cellular senescence [56]. NF-κB can be activated by many stimuli including oxidative stress, oncogenes, cytokines and DNA damage [56,57,58,59]. High Pi, both in vitro and in vivo, has been reported to induce VSMC calcification via mitochondrial reactive oxygen species mediated by RelA/p65 nuclear translocation which is an important subunit in the NF-κB complex [60].

### 4.2. Oxidative DNA Damage

ROS initiate lipid peroxidation, enormous damage to DNA (oxidative DNA damage) and proteins, and other abnormal biochemical changes [61]. Upon DNA damage, in order to maintain and restore genomic stability in response to an insult to the genome (for example induced by oxidative stress), mammalian cells utilise a complex signaling network to detect, signal, and eventually repair the encountered DNA damage [62]. If DDR mechanisms fail to repair the insult(s), then the damaged cell needs to be eliminated from the proliferating surrounding cells by cell death (apoptosis) or cell senescence (i.e. the arrest of normal cell division). Mounting evidence indicates that oxidative damage of DNA can be induced by ROS [63]. It is worth noting that oxidative DNA damage has been shown in atherosclerotic plaque VSMCs [64], which are already predisposed to calcification. The ERK1/2 pathway has been shown to be crucial in DDR and are known to increase the activity of ataxia telangiectasia-mutated protein (ATM) at the site of DNA damage [65]. Considering the well-documented regulatory role that Pi plays in modulating ERK signaling in VSMCs [41], it remains to be fully addressed whether Pi could promote VC via impairing ERK/ATM/DDR signaling leading to genomic instability. It also now merits establishing whether a high Pi milieu on VSMCs induces oxidative DNA damage in atherosclerotic plaques (which are already prone to calcification) and whether the persistent milieu concomitantly promotes such plaques to become calcified.

### 4.3. Cellular Senescence

VSMCs play an indispensable role in atherogenesis and regulation of VC. Meanwhile VSMC senescence is associated with atherosclerosis [66,67,68], aortic and arterial stiffness [69] and medial artery calcification [70]. Cellular senescence, the arrest of normal cell division, can be induced by a broad spectrum of cellular stressors or DNA damage. In human VSMCs, a higher extracellular Pi has been shown to induce cellular senescence via integrin linked kinase (ILK) overexpression dependent on IGF-1 receptor activation, and oxidative stress [71], which is a well-known inducer of DNA damage [63]. In addition, another study using kidney cells has shown that oxidative stress induces cellular senescence by increasing ILK protein expression and activity, which in turn reduces Klotho expression [72], indicating that Pi-induced cellular senescence via ILK might be applicable to multiple cell types. Klotho (⁻/⁻) mice show premature aging phenotypes and CKD-associated mineral and bone disorder (CKD-MBD)-like phenotypes mediated by hyperphosphatemia [73]. High Pi loading on human aortic SMCs has been shown to induce overexpression of p53, p21 and p16 and senescence-associated β-galactosidase (SA-β-gal) activity [71], further evidencing the emerging contribution of Pi-mediated VC by inducing cellular senescence.

### 4.4. Pi Regulation of VC via Modulating Intracellular Calcium Levels

Increases in intracellular calcium concentrations ([Ca^2+^]_i_), both as the result of an increase in store operated calcium entry (SOCE) and also impaired calcium extrusion from the cell, is critical for calcification of VSMCs [74]. Potassium limitation induces VSMC osteogenic differentiation and calcification by increasing [Ca^2+^]_i_ which itself enhances autophagy and promotes VSMC calcification by activating a cAMP response element–binding protein (CREB) signal [75]. In human aortic VSMCs, calcium-phosphate (CaPi) crystals potentiate cell death, an effect which has been shown to be a result of a CaPi-mediated increase in [Ca^2+^]_i_ and which can be reversed by treatment of cells with the lysosomal proton pump inhibitor bafilomycin A1 [44,76]. Nguyen NT et al [41] reported that in a sodium-dependant phosphate cotransport manner, high Pi results in an increase in [Ca^2+^]_i_; in turn, this rise in [Ca^2+^]_i_ could elicit oxidative stress and calcification of VSMCs. Using primary human aortic SMCs (HAoSMCs), Ma K and colleagues [77] reported that induction of osteo-/chondrogenic transdifferentiation of VSMCs and subsequent vascular calcification by Pi is mediated by upregulation of calcium release-activated calcium channel protein 1 (ORAI1) and stromal interaction molecule 1 (STIM1) expression and enhanced SOCE.

It is well-documented that high Pi induces a phenotypic change in VSMCs, resulting in the transdifferentiating of SMCs into osteoblast-like phenotypes, as evidenced by overexpression of Runx2 [78] and osteoblastic differentiation markers OSC and Cbfa-1 [40]. It has been shown that feeding nephrectomised rats a high phosphate diet even after a short time (two days) results in a significant increase in serum Pi and intact serum FGF-23 and PTH, as well as a significant decrease in ionised calcium [79]. PTH increases [Ca^2+^]_i_ levels, resulting in Ca^2+^ overload in platelets [80] and cardiomyocytes [81].

In VSMCs, increased calcium entry might affect their structure and function. A recent study looking at the effect of high Pi on VSMCs revealed that a high Pi milieu results in an increase in [Ca^2+^]_i_ [78,82]. This might partly explain why high levels of PTH (as seen in patients with renal insufficiency due to an increase in serum Pi) induces medial calcification. Arachidonic acid (AA) is involved in the regulation of [Ca^2+^]_i_ in a variety of cell types including endothelial cells [83,84] and underlying SMCs [85]. In mouse parotid acini it has been shown that inhibition of serine/threonine phosphatases enhances AA-induced ([Ca^2+^]_i_) via protein kinase A (PKA) [86]. A recent study has shown, in both calcified aortic valves and human valvular interstitial cells (VICs) under osteogenic induction, that PP2A activity is significantly decreased [49]. Pi is a potent inhibitor of a range of phosphoprotein phosphatases [28,29,30] including PP2A [28]. It remains to be determined if a high phosphate milieu on VSMCs induces calcification [5,16,40], mediated via increasing [Ca^2+^]_i_ in VSMCs by the action of Pi inhibiting phosphatases involved in regulation of [Ca^2+^]_i_ and mobility, which predisposes SMCs to calcification.

## 5. Role of Extracellular Vesicles in Mediating VC

In calcified arteries, apoptosis results in release of apoptotic bodies from VSMCs (similar structure to matrix vesicles which are known to be the nucleation sites for calcium crystal formation in bone) and may promote calcium crystal formation by acting as nucleating nodules [87]. Pi is well documented as the inducer for VSMC calcification and the effect of Pi on inducing calcification of VSMCs may be partly mediated by the effect of Pi in decreasing VSMC viability and apoptosis. In the A7r5 cell line (which is rat aorta origin cells), it has been reported that Pi induces calcification which is accompanied a decrease in cells’ viability [88]. In cultures of human VSMCs, it has been reported that calcium/phosphate crystals (CPC) in a size- and composite-dependent manner induce VSMC death and calcification, with smaller particle sizes (<1 µm in diameter) having greater potency to induce cell death and calcification [76]. In response to extracellular calcium, VSMCs release matrix vesicles (MVs) that compositionally are shown to be deferent in matrix Gla protein (as a crucial mineralization inhibitor) and on the contrary are enriched with matrix metalloproteinase-2 activity (MMP-2), annexin A2, A5, and A6, and phosphatidylserine (PSer) on the surface of MVs [89]. These molecules (Anexins and PSer) can enhance calcium binding, coagulation, and provide a suitable bases for hydroxyapatite nucleation and calcification. High extracellular Pi, both in vitro [29] and in vivo [48] in a rat partial nephrectomy model of CKD, has been reported to promote release of microvesicles expressing PSer and, hence, promote in vitro coagulation. Given the very well-known effect of extracellular Pi on VSMC dysfunction and the Pi-induced increase in [Ca^2+^]_i_ in VSMCs that has been discussed earlier, it is now remit to explore the composition of extracellular Pi-derived MVs from VSMCs and the effect of these vesicles on VSMC calcification, remodelling and coagulation.

## 6. Role of MicroRNAs in Regulating High Pi-Induced VC

miRs are small RNAs that negatively regulate gene expression via repression of the target mRNAs and are implicated to have substantial regulatory roles in pathologies such as CKD [90] and associated complications, including, notably, VC [91,92]. The role of miRs in VC is emerging even though it is well-documented that miRs play a substantial role in the pathophysiological mechanisms of VC [93]. Ets1 is an important transcription factor which is involved in enhancing VSMC remodelling through regulating genes encoding extracellular matrix proteins, such as OSP [94]. In primary cultured rat aortic VSMCs, it has been reported that treatment of cells with organic phosphate donor β-glycerophosphate decreased expression of miR-125b and its target, Ets1 [46]. It has been reported that high extracellular Pi load negatively regulated the expression level of miR-30b, miR-133a, and miR-143 which are involved in regulating Runx2, Smad1, and OSX [95].

Another study revealed that treatment of explanted rat aorta with extracellular Pi (6mM) resulted in altered expression of some miRs in a time-dependant manner, with up-regulation of miR-200c, -155, 322 and down-regulation of miR-708 and 331 at day three. Subsequently, this study found up-regulation of miR-328, -546, -301a and down-regulation of miR-409 and miR-542 after six days Pi-loading [91]. Furthermore, in response to high extracellular Pi levels, VSMCs experience increased proliferation and enhanced VSMC migration depending on Pi-induced upregulation of miR-223, which is an important marker of muscle damage and a key factor in osteoclast differentiation [96]. It remains to be determined whether high Pi-derived MVs may potentially carry the signature of such miRs and hence can promote vascular calcification elsewhere from their cells of origin. Given that hyperphosphatemia is a prominent mineral abnormality in CKD and incubating VSMCs with serum samples collected from patients with CKD has been shown to promote calcification in vitro [35,36], the potential, yet-to-be-discovered, existence of such Pi-derived miRs signatures, either free or within MVs, may partly explain the underlying mechanism of the observed CKD serum-induced calcification of cultured VSMCs.

## 7. Pi, Epigenetic and VC

Epigenetics play a crucial role in mediating calcification [97]. DNA methylation is one of the most important epigenetic mechanisms which is mediated by DNA methyltransferases (DNMTs) [98]. In response to high extracellular Pi levels, it has been shown that an increase in Pi influx induces phosphorylation and activation of the DNA methyltransferase (DNMT1) with subsequent association with the RAS protein activator-like 1 (RASAL1) promoter by histone deacetylase 2 (HDAC2), resulting in RASAL1 promoter cytosine phosphate-guanine (CpG) island hypermethylation which has been implicated in pathological endothelial–mesenchymal transition (EndoMT) in cardiac pathologies such as calcific aortic valve disease and cardiac fibrosis [99,100]. It has been reported that high Pi treatment of VSMCs both in vivo and in vitro dampens the expression of miR-34b which targets Notch1 which is involved in the process of calcification of VSMCs [101]. This effect of Pi-load in reducing the expression of miR-34b was shown to be due to Pi-induced hypermethylation of CpG island where the promoter of miR-34b is embedded. It has been further shown that the reported DNA hypermethylation and consequent miR-34b silencing was mediated by an increase in the expression of DNMT3a [101]. Extracellular Pi has also been reported to downregulate miR-204 (which targets DNMT3a) in calcified human renal arteries from patients with uremia, in calcified arteries of CKD mice receiving a high-phosphate diet, and in cultures of VSMCs [102]. Given the substantial regulatory role of high Pi in enhancing cellular protein phosphorylation signals [28,29], of note phosphorylation and activation of DNMT1 [99], and a recent report highlighting the emergence of the epigenetic regulatory role that Pi may play in regulating VC by augmenting miR-34b expression downstream of CpG island hypermethylation, it now merits further investigation whether Pi is involved in transdifferentiation and calcification of VSMCs by altering phosphorylation status and hence activity of DNMTs in VSMCs and their potential, yet-to-be-discovered, targeted miRs involved in regulating VSMCs remodelling and calcification.

## 8. Conclusions and Directions for Future Work

VC is a complex and highly regulated process. Over the past two decades, tremendous progress has been made in our understanding of the regulation of VC. However, despite this progress, more work is needed to determine the precise mechanisms underlying the initiation and progression of VC in different disease settings, particularly in CKD. VSMCs promote mineralization by mechanisms involving osteo-/chondrogenic transdifferentiation. Pi-induced transdifferentiation of VSMCs to an osteo-/chondrogenic phenotype is controlled by complex intracellular signaling pathways. Given the crucial role that Pi plays in regulation of VSMCs’ phenotypical transdifferentiation to bone-like cells and VC, as well as the emergence of evidence shedding light on the possible interplay that Pi may have in promoting calcification by acting via epigenetic pathways, regulation of [Ca^2+^]_i_, defective DDR and cellular senescence, there is a need for further studies to clarify precise signaling molecules and targets to limit initiation or mitigate the progression of calcification in patients at high risk of developing VC, such as those with CKD.

## Data Availability

Not applicable.
